# Damp Walls

**Published:** 1877-07

**Authors:** 


### DAMP WALLS.

Multitudes of people contemplate building family dwellings this year. Most persons can bring to their remembrance cases where splendid mansions have been erected with a portion of the wealth which a life-time of well directed industry and economy has secured, and just about the time when everything has been completed, the owner has lain down and died; if not indeed, other members of the family; damp walls are a sufficient, yet not the only cause of such a result. Walls are not damp of themselves, but they are made so, as a pane of glass is made damp, the glass itself being colder than the atmosphere of the room, condenses some of the moisture which that atmosphere contains, and drops of water are formed on its surface; a glass or pitcher of ice water presents the same appearance. In southern cities, streams of water may be seen on the floor, having trickled down from the walls when the atmosphere has been overcharged with vapor. To prevent this, strips of wood an inch or more thick, should be fastened to the walls, on which the laths should be nailed, this leaves a space for the circulation of the air, and keeps the whole building dry in all seasons of the year. Our readers may rest assured, that a very large proportion of the diseases which afflict men and prevent them living *out half their days* literally, arise from ignorance, and inattention to the known laws of things.

---

HAIR, or even STRAW MATTRESSES, are more healthy to sleep on than feather beds. Never put children on these heating beds. Keep their sleeping rooms very clean and well-aired, and do not cumber them with unnecessary furniture.

